# Dihydrotanshinone-I interferes with the RNA-binding activity of HuR affecting its post-transcriptional function

**DOI:** 10.1038/srep16478

**Published:** 2015-11-10

**Authors:** Vito Giuseppe D’Agostino, Preet Lal, Barbara Mantelli, Christopher Tiedje, Chiara Zucal, Natthakan Thongon, Matthias Gaestel, Elisa Latorre, Luciana Marinelli, Pierfausto Seneci, Marialaura Amadio, Alessandro Provenzani

**Affiliations:** 1 Centre For Integrative Biology (CIBIO), University of Trento, Trento, 38123, Italy; 2 Department of Biochemistry, Hannover Medical University, Hannover, D-30625, Germany; 3 Department of Pharmacy, University of Naples “Federico II”, Naples, 80131, Italy; 4 Chemistry Department, University of Milan, Milan, 20133, Italy; 5 Department of Drug Sciences, University of Pavia, Pavia, 27100, Italy

## Abstract

Post-transcriptional regulation is an essential determinant of gene expression programs in physiological and pathological conditions. HuR is a RNA-binding protein that orchestrates the stabilization and translation of mRNAs, critical in inflammation and tumor progression, including tumor necrosis factor-alpha (TNF). We identified the low molecular weight compound 15,16-dihydrotanshinone-I (DHTS), well known in traditional Chinese medicine practice, through a validated high throughput screening on a set of anti-inflammatory agents for its ability to prevent HuR:RNA complex formation. We found that DHTS interferes with the association step between HuR and the RNA with an equilibrium dissociation constant in the nanomolar range *in vitro* (Ki = 3.74 ± 1.63 nM). In breast cancer cell lines, short term exposure to DHTS influences mRNA stability and translational efficiency of TNF in a HuR-dependent manner and also other functional readouts of its post-transcriptional control, such as the stability of selected pre-mRNAs. Importantly, we show that migration and sensitivity of breast cancer cells to DHTS are modulated by HuR expression, indicating that HuR is among the preferential intracellular targets of DHTS. Here, we disclose a previously unrecognized molecular mechanism exerted by DHTS, opening new perspectives to therapeutically target the HuR mediated, post-transcriptional control in inflammation and cancer cells.

Post-transcriptional control of messenger RNA, coordinated by RNA-binding proteins (RBPs) and small or long non-coding RNAs, is an essential determinant of protein expression. Altered mRNA stability of pro-inflammatory cytokines tightly correlates with several pathological conditions such as inflammation, autoimmune disorders and cancer^1^. A prominent example of cytokine subjected to post-transcriptional control is tumor necrosis factor alpha (TNF-alpha or TNF), one of the main mediators of chronic inflammation associated with malignant cell transformation, growth and tumor progression^2^. Depletion of several RBPs can alter TNF protein production, leading to exacerbated chronic inflammatory disease both in mice and in humans^3^, supporting the relevance of *in vivo* post-transcriptional control on TNF mRNA. The half-life of this transcript is influenced by competitive binding of RBPs to adenylate- and uridinylate-rich elements (AU-rich elements or AREs) and by a constitutive decay element (CDE) in its 3′-untranslated region (UTR)^4^,^5^. Notably, it has been shown that the stability and translational efficiency of TNF mRNA is dependent on the p38 MAPK pathway, whose activation modulates the cytoplasmic equilibrium of tristetraprolin (TTP or Zfp36) and HuR/ELAVL1 proteins^6^. Whilst TTP is an anti-inflammatory RBP favoring rapid mRNA degradation, HuR stabilizes transcripts and promotes their poly-ribosomes engagement for active translation. This post-transcriptional function of HuR has been described for a wide number of transcripts bearing AU-rich elements, whose turnover is critical for cell proliferation, tumor cell survival, angiogenesis and metastasis^7^,^8^,^9^,^10^. Sporadically, anti-inflammatory agents have been reported to post-transcriptionally modulate cytokines, including TNF, with a variable involvement of the p38 MAPK pathway, as in the case of KL-1037^11^, s-curvularin^12^, LCY-2-CHO^13^. However, the direct and specific modulation of defined *trans*-acting factors has remained elusive. On the other side, systematic investigations, based on the direct evaluation of a post-transcriptional read-out have shown the feasibility of considering RBPs as potential drug targets^14^,^15^,^16^. Interestingly, resveratrol was found to suppress activation-induced gene expression in T-cell via a post-transcriptional mechanism and its effects were rescued by the RBP HuR. The same molecule was found to exert its post-transcriptional effects by regulating the RBP KSRP, suggesting that resveratrol changes the mRNA stability of HuR-targeted transcripts by enhancing the 3′-UTR binding of KSRP and replacing HuR from the same 3′-UTR sequences^16^. Here we demonstrate that 15,16-dihydrotanshinone-I (DHTS), identified through a biochemical high-throughput screening among a set of anti-inflammatory agents, inhibits the HuR-RNA complex formation *in vitro* in low nanomolar range. DHTS belongs to the bioactive family of diterpenic tanshinones, extracted from the roots of *Salvia miltiorrhiza* and well-known in traditional Chinese medicine practice. Tanshinones are anti-inflammatory agents used for treatment of cardiovascular diseases^17^ and during the last years they have been proposed as anti-cancer agents due their anti-proliferative, anti-angiogenic and pro-apoptotic activities against a broad spectrum of tumors^18^,^19^. We provide evidences that interference of DHTS on HuR activity determines a post-transcriptional influence of TNF mRNA processing, showing a previously unrecognized molecular mechanism for this class of small molecules. In addition, we show that cytotoxicity and migration properties of breast cancer cell lines treated with DHTS are influenced by HuR dosage, supporting the post-transcriptional effect of this compound as a new, therapeutically relevant molecular mechanism.

## Results

### 15,16-Dihydrotanshinone I (DHTS) interferes with HuR-RNA interaction *in vitro*

**Figure 1:**
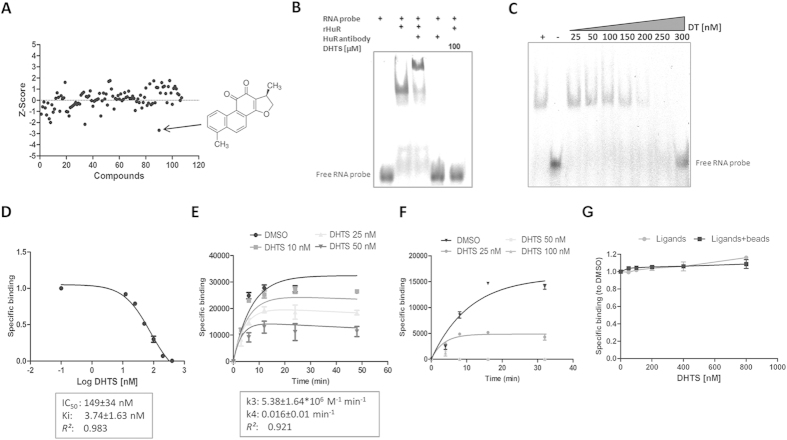
DHTS is an inhibitor of the rHuR-RNA interaction. (**A**) AlphaScreen HTS carried out using 1 nM of rHuR, 50 nM of BiTNF RNA probe and 50 nM of 107 anti-inflammatory compounds (see Table S1). (**B**) Representative REMSA performed with 0.5 μM of rHuR and 0.5 μM of Cy-3 RNA probe at equilibrium, showing the inhibitory activity of DHTS and its un-efficacy to electrophoretic mobility of the free RNA even at 100 μM. (**C**) Saturation binding by REMSA or (**D**) by AlphaScreen assays evaluating DHTS activity in low micromolar or nanomolar range, respectively. (**E**) Kinetic experiments showing association (k3) and dissociation (k4) rate constants of DHTS. (**F**) Kinetic experiments performed upon pre-incubation of 1 nM of rHuR with different concentrations of DHTS before addition of 50 nM of BiTNF probe. (**G**) Dissociation experiments performed upon 30 min pre-incubation of 1 nM of rHuR and 50 nM of RNA (Ligands), or 30 min pre-incubation of Ligands+beads, before addition of DHTS. Mean ± SD refers to three independent experiments (n = 3).

**Figure 2:**
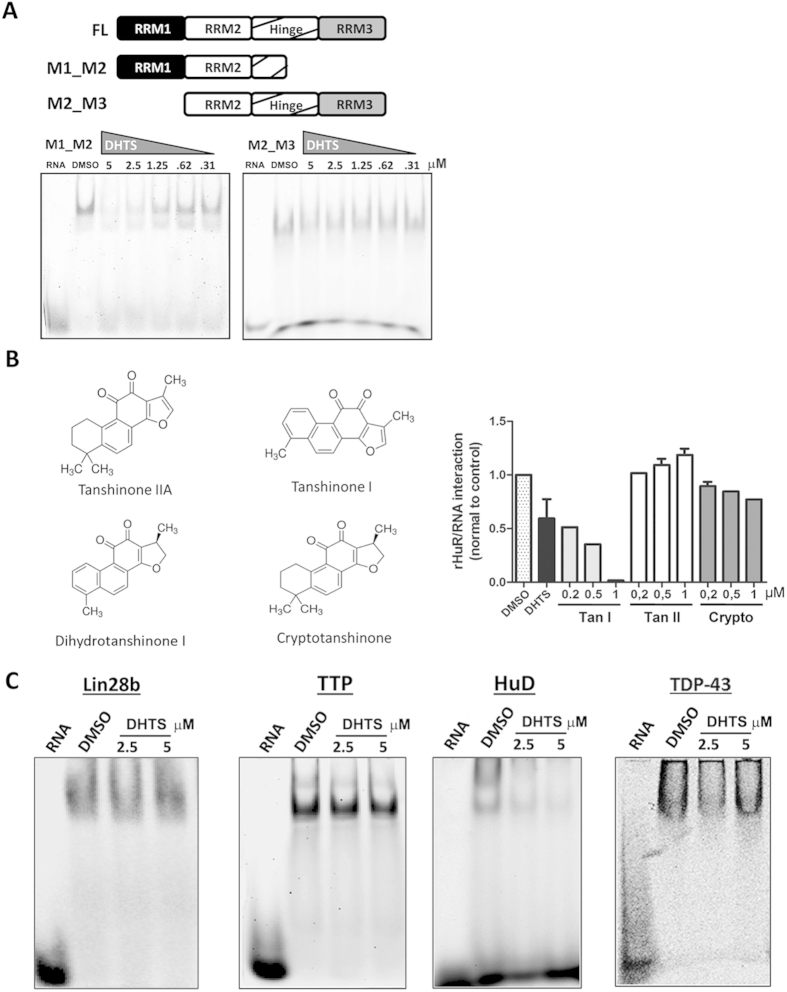
DHTS inhibits HuR’s first RRMs and is not effective against other RBPs, HuD excluded; *in vitro* activity of other tanshinones. (**A**) Representative gels of at least three independent protein preparations of recombinant M1_M2 and M2_M3 HuR proteins. REMSAs were performed with 0.5 μM of protein, 0.5 μM of Cy-3 RNA probe and DMSO or DHTS at indicated doses. (**B**) Quantification of specific HuR-RNA binding challenged by indicated concentrations of commercially available tanshinones, including cryptotanshinone; TanI and TanII that were not included in the first screening. (**C**) Evaluation of DHTS activity at the indicated concentrations against Lin28b, TTP, HuD, and TDP-43 RNA-binding proteins tested by REMSA with 0.5 μM of Cy-3 RNA as indicated in methods. Mean ± SD refers to three independent experiments (n = 3).

The mRNA stabilizing effects of HuR have been frequently investigated by studying the post-transcriptional control of key pro-inflammatory genes like *COX-2*, *TNF* or specific cytokines such as *IL17*^20^,^21^,^22^. We hypothesized that anti-inflammatory small molecules could integrate a post-transcriptional layer of influence on mRNAs, in their mechanism of action, by interfering with HuR function. By using a previously validated biochemical approach^23^ involving the 3′UTR ARE of TNF as RNA probe and a recombinant human HuR protein, we screened a set of commercially available anti-inflammatory compounds (a total of 107 listed in Supplementary Table S1) for their ability to prevent the rHuR-RNA complex formation. Eight positive hits were obtained (DHTS, hydrocortisone acetate, amiprilose, flurbiprofen, deracoxib, fluocinolone, triamcinolone, dexamethasone). The most potent hit, DHTS (CHEMBL227075 ID in ChEMBL database, Z-Score = −2.69, Fig. 1A and Supplementary Table S1), among the other compounds, confirmed this inhibitory activity in RNA electrophoresis mobility shift assays. DHTS did not alter the electrophoretic mobility or the stability of the RNA probe even when a 200 fold molar excess (100 μM) was used in the assay (Fig. 1B and Supplementary Fig. S1A), behaving as a validated hit. Saturation binding competitive experiments at equilibrium, either by REMSA (Fig. 1C) or AlphaScreen assays (Fig. 1D), indicated an equilibrium dissociation constant for DHTS (K_i_) of 3.74 ± 1.63 nM, using a K_d_ equal to 2.5 nM^23^, and IC_50_ equaling 149 ± 34 nM and 68 ± 16 nM for REMSA or AlphaScreen assays, respectively and according to the different concentration of the reagents used. The range of K_i_ values was also confirmed by competitive binding kinetics experiments (Fig. 1E), that revealed an association rate constant (k3) of 5.38 ± 1.64*10^6^ M^−1^ min^−1^ and a dissociation rate constant (k4) of 0.016 ± 0.01 min^–1^ for DHTS (k4/k3 = 2.97 ± 0.7 nM). From these data, representing the law of mass action parameters of DHTS, it can be inferred that DHTS has a much higher probability to associate with one or both the free ligands, rather than to displace a pre-formed protein-RNA complex. Consistently, binding kinetic experiments performed either upon pre-incubation of rHuR with different concentrations of DHTS (Fig. 1F), or upon pre-incubation of protein and RNA (Fig. 1G), provided evidence that DHTS mainly prevents the association between the two ligands. Since the recognition of RNA substrate is determined by the RNA recognition domains (RRMs) of HuR, we purified two recombinant HuR isoforms (Supplementary Fig. S1B) retaining two different arrangements of RRMs, as depicted in Fig. 2A. As RRM1 and RRM2 are the rate-limiting domains for HuR binding activity^24^, the M1_M2 construct was expected to best recapitulate the ability of full-length HuR to bind to RNA, and to be the best target for DHTS. Accordingly, at equilibrium, M1_M2 isoform showed a K_d_ value (2.66 nM) similar to full-lenght HuR. DHTS similarly inhibited the M1_M2-RNA complex formation (K_i_ = 4.12 ± 0.81 nM). Notably, the M2_M3 construct expressed a labile protein characterized by higher K_d_ value (~24 nM) and only limited effects of DHTS were observed. In addition, we purified each single RRM domain (M1, M2, M3) from E. coli cells (Supplementary Fig. S1C) and analyzed their binding to the RNA probe in the presence of DHTS. The binding of M2 and M3 was not affected even using 10 μM of DHTS, while M1 was only marginally affected, being 74% of the protein still binding RNA with 5 μM of DHTS (Supplementary Fig. S1C). Taken together, these results suggest that the compound interferes with RNA binding by affecting a different site from the domain of RNA recognition. A putative explanation could reside in the interference by DHTS with the allosteric conformational changes of the first two RRMs, disturbing the additional contacts formed with the inter-domain linker region during RNA binding^24^. The *in vitro* activity of three commercially available members of the tanshinone family of compounds was evaluated in our biochemical model. Each tested tanshinone showed inhibitory activity (Supplementary Fig. S1D). However, while cryptotanshinone and tanshinone IIA were less potent (micromolar range), the potency of tanshinone I was comparable to DHTS at equilibrium (Fig. 2B). This indicates how this kind of interference requires either an aromatic furan ring (as in tanshinone I), or a reduced dihydrofuran (as in DHTS) on the right portion of the molecule. Conversely, the left side of tanshinones must contain a planar, aromatic methyl-substituted ring (as in DHTS and tanshinone I), rather than a non-planar, dimethyl-substituted cyclohexene ring (as in cryptotanshinone and tanshinone IIA). As tanshinone I is poorly soluble in buffers of biochemical assays (producing visible precipitates at the highest doses), we used DHTS as reference compound/inhibitor to exploit this bioactivity for further experiments.

Albeit the selective profile of DHTS and tanshinones has to be systematically characterized, we selected four other RBPs, i.e. Lin28b, TTP, TDP-43 and ELAVL4/HuD, with different structural similarity compared with HuR and tested DHTS as a modulator of their specific protein-RNA interactions (Fig. 2C). As expected considering the 78% structural similarity with HuR, only HuD-RNA complex formation was affected by DHTS at the reference doses. Conversely, the binding of Lin28b, TTP, and TDP-43 to RNA did not appear substantially affected. Taken together, these biochemical data allowed to quantitatively determine the DHTS-mediated inhibition of the HuR-RNA complex formation *in vitro*, the highest potency of DHTS among the tested analogs and its selective profile towards other RBPs. Then, we decided to deeply investigate the post-transcriptional role of DHTS in a panel of breast cancer cell lines, characterized by HuR over-expression^25^.

### DHTS down-regulates TNF mRNA and protein levels

**Figure 3:**
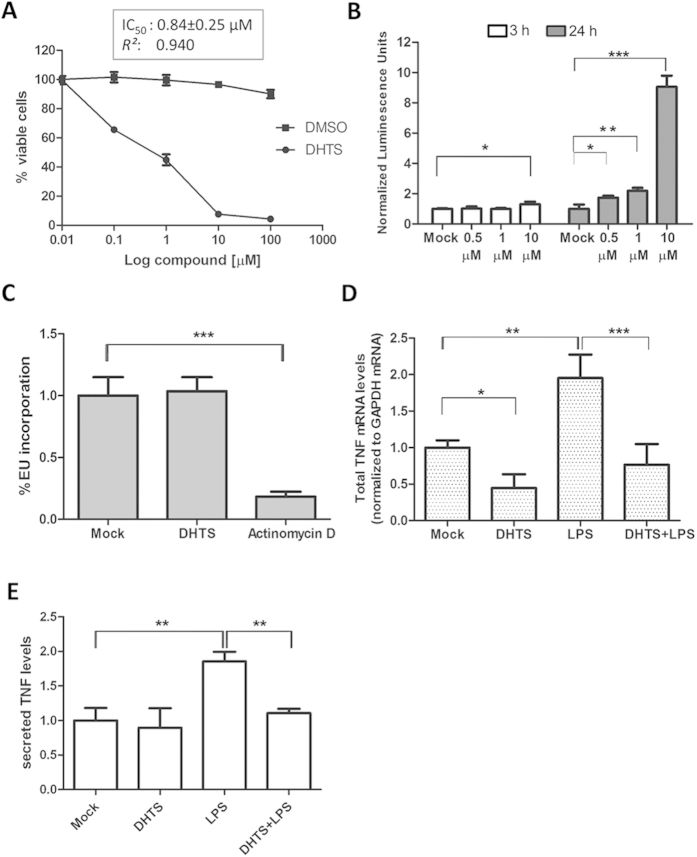
DHTS toxicity and inhibition of TNF in MCF-7 cells. (**A**) CellTiter-Glo assays upon exposure of MCF-7 cells to DHTS for 24 h. Relative IC_50_ was calculated by nonlinear regression curve fitting. (**B**) Apoptosis evaluation by 3/7 Caspase-Glo luminescent assays (Promega) and normalization to trypan blu negative cells (n = 3). (**C**) High content imaging quantification of fluorescence intensity/cell population of EU-conjugated Alexa-488 after 3 h treatment of MCF-7 cells with 1 μM of DHTS or 2 μM of actinomycin D. (**D**) Q-RT-PCR of TNF mRNA levels. Relative abundance was normalized with GAPDH mRNA in MCF-7 cells. (**E**) ELISA measuring secreted TNF protein levels. We detected in Mock condition an average of 15 pg/ml of sTNF as obtained by titration with standards. Where not indicated, mean ± SD refers to four independent experiments (n = 4).

Viability assays measuring intracellular ATP levels in different breast cancer cell lines (MCF-7, MDA-MB-231 and SKBR3), showed that about 50% of cells were not viable after 24 h treatment with 1 μM DHTS (MCF-7, IC_50_ = 0.84 μM, Fig. 3A; MDA-MB-231, IC_50_ = 0.92 μM, and SKBR3, IC_50_ = 1.2 μM, graphs not shown). In MCF-7 cells, caspase activation was clearly visible after 24 h of treatment using as low as 0.5 μM of DHTS. Conversely, at an earlier time point (3 h), no caspase activation was observed with 1 μM of DHTS (Fig. 3B) and total RNA was actively transcribed (Fig. 3C and Supplementary Fig. S2A). Therefore, we used 1 μM DHTS treatment at 3 h as reference condition to further characterize the impact of DHTS on HuR activity, and to reasonably exclude the induction of dramatic molecular events associated with cytotoxicity of the compound. DHTS alone or in combination with 3 h LPS stimulation (100 ng/ml) reduced TNF levels in stimulated mouse RAW264.7 cell line (Supplementary Fig. S2B)^26^. Since chronic production of TNF influences the phenotype of breast cancer cells by inducing secondary cytokines production and by impacting their growth and metastatic potential^27^, we studied the effect of DHTS on the regulation of TNF in human breast cancer cell lines. Exposure of breast cancer cells to 1 μM of DHTS for 3 h significantly reduced total TNF mRNA levels to ≈40% (P < 0.05) of the control levels (Fig. 3D and Supplementary Fig. S2C). Treatment with 1 μg/ml of LPS to MCF-7 (TLR4 positive) enhanced the TNF mRNA levels (P < 0.01), while treatment with DHTS counteracted the LPS-induced up-regulation (P < 0.001) (Fig. 3D). MCF-7 cells expressed almost undetectable levels of TNF protein, and the endogenous (pro-TNF) protein could not be detected by immunoblotting in standard conditions. However, massive stimulation of pro-TNF production *via* exposure of MCF-7 cells to *E. coli* cells, and subsequent administration of DHTS, showed the efficacy of the compound in attenuating the production of pro-TNF (Supplementary Fig. S2D). Similarly, endogenous secreted TNF (sTNF, Fig. 3E) was hardly detectable under basal conditions, but protein level increased after LPS treatment and decreased markedly after 3 h treatment by DHTS (P < 0.01). These data show that DHTS first reduces the TNF mRNA expression level, and subsequently the translation and secretion of the encoded protein, confirming the anti-inflammatory properties of the compound in these cellular models.

### DHTS inhibits post-transcriptional effects of HuR in MCF-7 cells

**Figure 4:**
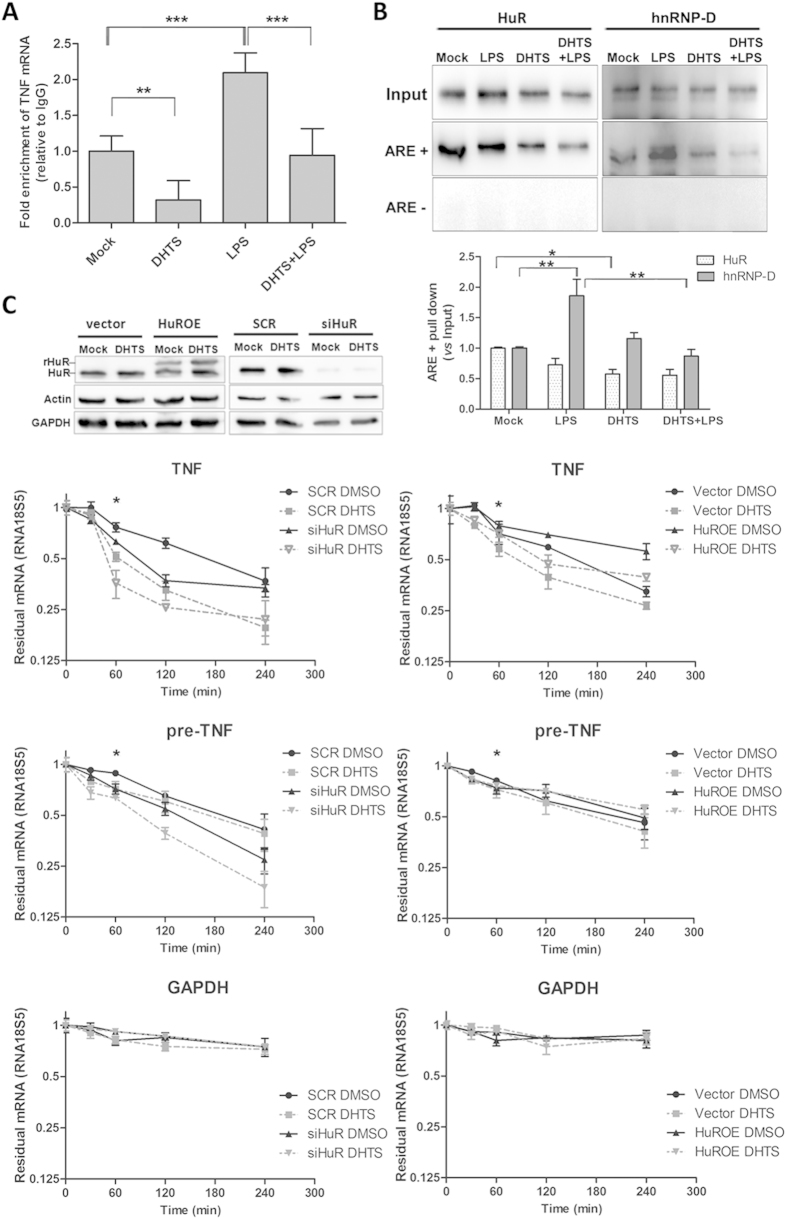
DHTS inhibits intracellular HuR-mRNA association, influencing mRNA stability. (**A**) RNA immunoprecipitation using MCF-7 lysates obtained after 3 h of treatment with DMSO, 1 μM of DHTS, 1 μg/ml of LPS, or DHTS+LPS co-treatment. (**B)** RNA pull down assays on MCF-7 lysates obtained as in (**A**) after 2 h incubation with BiTNF (ARE+) or BiTNFneg (ARE-) exogenous RNA probes. Graph shows densitometric analyses by Image J software (NIH). (**C**) mRNA stability evaluation of TNF, pre-TNF, and GAPDH after co-treatment with actinomycin (**D**) and DHTS of scramble, vector, HuR silenced (siHuR) and HuR over-expressing (HuROE) MCF-7 cells. Relative HuR expression levels are shown in the representative WB. Residual mRNA, plotted in log scale, was normalized to relative RNA18S5 mRNA levels. Mean ± SD refers to three independent experiments (n = 3).

To test whether DHTS influences the post-transcriptional control of TNF mRNA mediated by HuR we performed ribonucleoproteic immunoprecipitation (RIP) analyses. DHTS reduced the number of copies of TNF mRNA selectively bound by HuR in MCF-7 cells (Fig. 4A). The strong enrichment observed in LPS-stimulated cell extracts confirmed the functional role of HuR in the post-transcriptional control of TNF in human cancer cells (P < 0.001). Notably, this effect is not specific for TNF mRNA, since RIP analysis on other HuR regulated transcript demonstrated that the copy number of ERBB2, VEGF, and CCND1 mRNAs bound by HuR in the presence of DHTS is, with different extent, significantly less with respect to the control (Supplementary Fig. S3A). RNA pull-down experiments in lysates from DHTS-treated MCF-7 cells confirmed the inhibition of the association step leading to the HuR:RNA complex formation (Fig. 4B), being the differences of precipitated HuR protein in DHTS treated cells statistically significant (P < 0.05) comparing DHTS *vs* Mock using the biotinylated probe. The same trend was observed for DHTS+LPS *vs* LPS condition, although with a non-significant effect in these experiments. Another ARE-binding RBP, hnRNP-D/AUF1, also displayed differential RNA-binding activity, however we observed these differences exclusively upon stimulation with LPS. To understand whether DHTS influences the stability of TNF mRNA in a HuR-dependent manner, we used both a stable HuR-silenced MCF-7 clone (siHuR) and transiently HuR over-expressing MCF-7 (HuROE) cells in actinomycin D chase experiments (Fig. 4C). The expression level of HuR positively correlated with the relative abundance of total TNF mRNA in MCF-7 cells, upon simultaneous transcriptional block. Interestingly, DHTS significantly reduced TNF mRNA stability compared with mock cells (P < 0.01 at 60 min time point), and caused a slightly additive effect in siHuR cells. On the contrary, DHTS displayed less efficacy in HuROE cells, suggesting that HuR expression is able to counteract the destabilizing effect of DHTS on mature TNF mRNA. To better understand these functional relationships, we evaluated the stability of nuclear immature (pre-) TNF transcripts, confirming, although with different kinetics, the HuR-dependency of mRNA stability and the same effects induced by DHTS on mature RNA transcripts; Similar effects were not observed for GAPDH mRNA under the same experimental conditions (Fig. 4C). Given the emerging evidences regarding HuR functionality in pre-mRNA processing events^28^, candidate HuR pre-mRNA targets were chosen according to Mukherjee *et al.*^28^, and CD14 was chosen as a negative control lacking AREs^14^. The mRNA stability of individual mRNAs, in actinomycin D chase experiment at single time point, was differently regulated by HuR expression and, except for pre-CTCF mRNA, HuR silencing caused a reduction of pre-BRCA1, pre-MDM2, pre-MYBL2 and pre-NFATC3 mRNA stability (Supplementary Fig. S3B, compare bar 1 and 3), in agreement with reported data^28^. To a different extent, DHTS diminished the stability of all the pre-mRNAs except the non-target, ARE-lacking, CD14 pre-mRNA (Supplementary Fig. S3B, compare bars 1 to 2). The compound exhibited further destabilization of pre-mRNAs in HuR depleted cells (siHuR), with the exception of pre-NFATC3 mRNA (Supplementary Fig. S3B, compare bars 3 to 4 and 5 to 6). Strikingly, over-expression of HuR in HuROE cells rescued the destabilization effect of DHTS to control levels (Supplementary Fig. S3B, compare bars 7 and 8), supporting the idea that HuR RNA-binding activity is a target of DHTS, and that this compound early impacts this post-transcriptional modulation.

### DHTS reduces polysomal recruitment of cytoplasmic TNF mRNA

**Figure 5:**
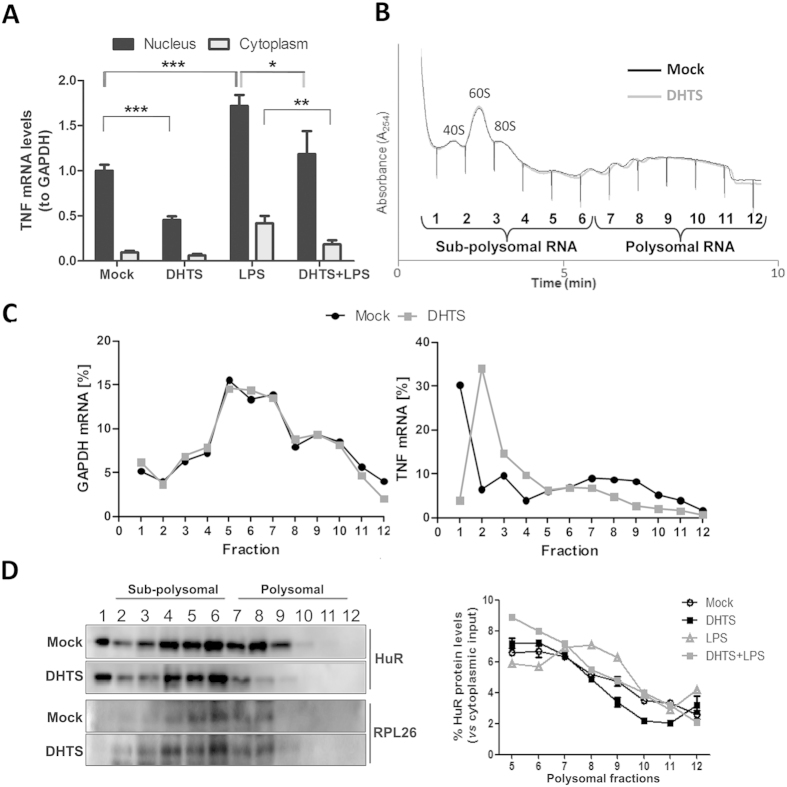
DHTS decreases TNF mRNA translational efficiency in MCF-7 cells. (**A**) Q-RT-PCR showing relative nuclear or cytoplasmic amounts of TNF mRNA after 3 h treatment, normalized with GAPDH mRNA levels. (**B**) Polysomal profiles of cytoplasmic RNA of MCF-7 treated for 3 h with DMSO or 1 μM of DHTS. (**C**) Q-RT-PCR analysis of GAPDH and TNF mRNA levels in single cytoplasmic RNA fractions. (**D**) Representative WB showing the distribution of HuR and RPL26 ribosomal protein in single fractions (left); densitometric analysis of relative cytoplasmic HuR protein levels in polysomal fractions. Mean ± SD refers to three independent experiments (n = 3).

To evaluate if DHTS modifies the recruitment of TNF mRNA to the translational machinery, we performed nuclear and cytoplasmic RNA purifications from MCF-7 cells treated with DHTS and/or LPS, respectively, and evaluated TNF mRNA levels by real-time PCR (Fig. 5A). Nuclear levels of the transcript were significantly reduced by DHTS both in case-control and in LPS-treated cells (of ~55% and ~31%, P < 0.001 and P < 0.05, respectively). The relative amount of cytoplasmic TNF mRNA was unchanged in DHTS-treated versus control cells, but was significantly reduced by DHTS treatment (~44%, P < 0.01) in LPS-stimulated cells. Therefore, DHTS down-regulated nuclear TNF mRNA and reduced cytoplasmic TNF mRNA only after LPS stimulation.

**Figure 6:**
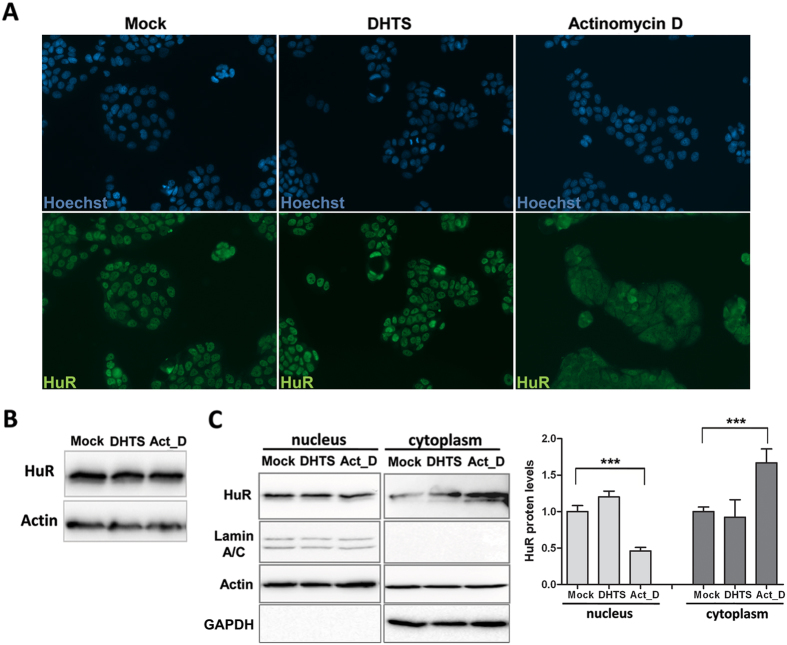
Effect of DHTS on HuR sub-cellular localization. (**A**) Representative immunofluorescence showing nuclei (Hoechst) or endogenous HuR (green) in MCF-7 cells treated with 1 μM DHTS or 2 μM of Actinomycin for 3 hD. (**B**) Representative western blot of total protein levels of HuR. (**C**) Western blot analysis on nuclear and cytoplasmic extracts of MCF-7 treated as in A. Densitometric analysis plot data of three independent experiments (n = 3).

To functionally investigate the translational efficiency of TNF mRNA, we performed sucrose-gradient fractionations of cytoplasmic sub-polysomal (representative of non-translating monosomes) and polysomal RNAs (representative of actively translating poly-ribosomes). DHTS treatment was performed on MCF-7 cells as such (Fig. 5B), and on LPS-treated MCF-7 and RAW264.7 cells (Supplementary Fig. S4A, S4B, S4C). Overall polysomal profiles showed no qualitative differences between the conditions analyzed, but the distribution analysis of single mRNAs, i.e. GAPDH and TNF (Fig. 5C) demonstrated that treatment with DHTS clearly reduced the polysomal loading of TNF mRNA paralleled by an increased amount in the sub-polysomal fractions. These data are also supported by the quantification of the TNF mRNA in collected sub-polysomal (fraction 1 to 6) and polysomal (fraction 7 to 12) compartments (Supplementary Fig. S4B). Notably, the effect of DHTS on poly/sub ratios increased significantly with concomitant LPS stimulation (P < 0.001). Accordingly, polysomal loading of mouse TNF mRNA was also affected by DHTS in stimulated RAW264.7 cells (Supplementary Fig. S4C), supporting the hypothesis that DHTS interferes with the cytoplasmic TNF mRNA fraction loaded on polysomes for its translation. Extensive literature data show that HuR enhances the stability and translation of its bound mRNA by favoring the polysomal recruitment of the transcript^21^. Western blot analyses on protein samples precipitated from polysomal fractions revealed that DHTS displaces HuR from heavy polysomal fractions in un-stimulated and LPS-stimulated cells (Fig. 5D). Notably, we could not detect alterations in the activation of the p38 MAPK pathway in RAW264.7 cells (Supplementary Fig. S4D), which is responsible for cytoplasmic re-localization of HuR and stabilization of TNF mRNA upon LPS stimulation^6^. Similarly, treatment of 3 h with 1 μM of DHTS did not affect sub-cellular localization of HuR in MCF-7 cells (Fig. 6A) and did not change HuR protein expression level (Fig. 6B). Nucleo-cytoplasmic fractionation confirmed DHTS-induced HuR nuclear localization (Fig. 6C) further supporting the loss of function of HuR in the cytoplasm and its reduced polysomal loading. Our data suggests that, at early time points, pharmacological inhibition of RNA loading on HuR by DHTS confines HuR into the nuclear compartment.

### HuR expression influences the sensitivity of breast cancer cells to DHTS

**Figure 7:**
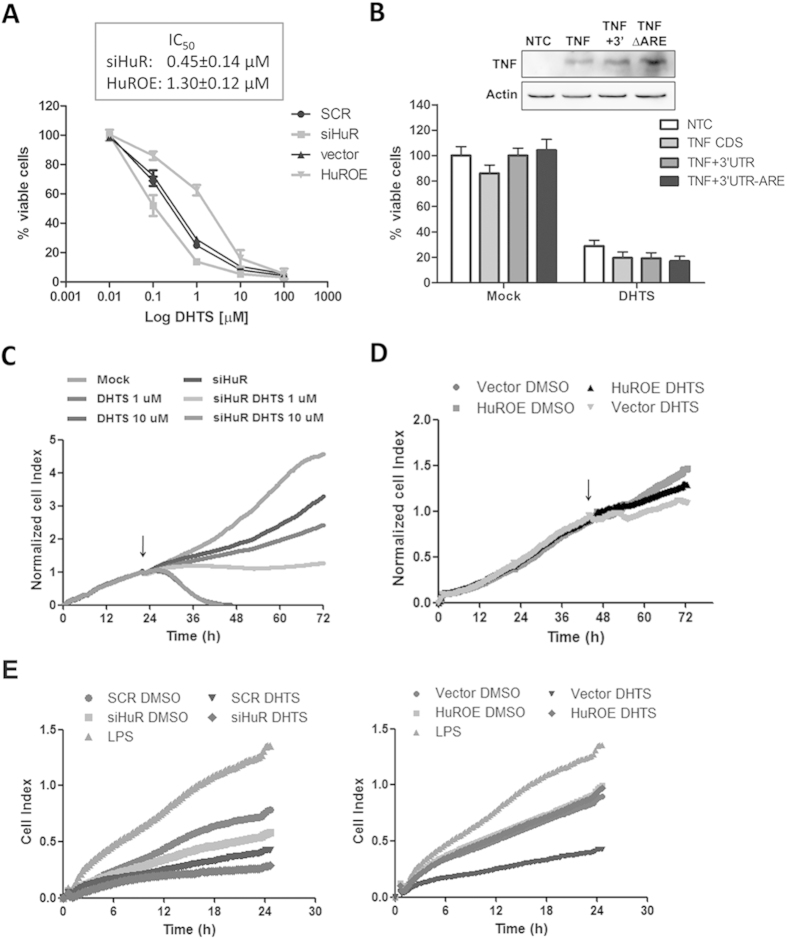
Efficacy of DHTS is dependent on HuR in breast cancer cell lines. (**A**) MTT assays on MCF-7 cells genetically ablated or over-expressing HuR and treated for 24 h with DMSO or DHTS. (**B**) MTT assays on MCF-7 un-transfected or transfected with pUNO1-hTNFA (TNF), pUNO1-hTNFA/3′UTR (TNF+3′), or pUNO1-hTNFA/3′UTR-ARE (TNFΔARE) plasmids, then treated as in A. Western blot shows relative amount resulting from ectopic expression of TNF. (**C,D**) RTCA proliferation assays. Arrows indicate the treatment point with DMSO or the indicated doses of DHTS (symbol of vector DMSO condition is behind HuROE DHTS in the figure). (**E**) RTCA migration assays. Complete media of SCR, vector, siHuR, and HuROE MCF-7 cells treated for 3 h with DMSO, 1 μM of DHTS 1 μg/ml of LPS (vector cells only for positive control) were diluted to obtain 1% FBS final concentration. MDA-MB-231 cells were equally seeded (20,000/well) in each well of the upper chamber. SCR; stably transfected cells with non targeting shRNA, siHuR; stably transfected cells with HuR targeting shRNA, vector; transient transfected cells with empty vector, HuROE; transient transfected cells with HuR expressing vector. Mean ± SD refers to three independent experiments (n = 3).

Dose-response assays on HuR-silenced or -over-expressing MCF-7 cells revealed a different sensitivity to DHTS (IC_50_ at 24 h of 0.45 or 1.3 μM compared with scramble or vector MCF-7 cells, respectively) (Fig. 7A), showing a compensatory effect of HuR against DHTS. Interestingly, ectopic expression of TNF CDS alone, with 3′UTR and ARE or with 3′UTR but without ARE, did not influence the sensitivity of cells to 1 μM of DHTS (Fig. 7B). This indicates that TNF itself is not responsible for this phenotypic response and that it can depend on the dysregulation of other factors regulated by HuR or, also, on independent events triggering the apoptotic pathways (Figs 3B, 7A). Real-time cell analysis assays demonstrated that DHTS exerts anti-proliferative effects at low doses (1 μM), in MCF-7 cells after about 12 h of treatment (Fig. 7C). At higher doses (10 μM) cytotoxic effects of the compound appeared in this experimental system, and were confirmed by MTT (data not shown). HuR-silenced MCF-7 cells showed a decreased proliferative rate, and DHTS treatment completely blocked cell proliferation. Conversely, the proliferative potential of HuR over-expressing cells was considerably influenced by 1 μM of DHTS compared with control (Fig. 7D), indicating that HuR dosage can modulate phenotypic response of these cells to low DHTS doses.

To better understand the phenotypic effect related with down-regulation of HuR-dependent and/or signaling molecules such as TNF, we then investigated the chemotactic potential of DHTS-treated, MCF-7 conditioned medium using MDA-MB-231 trans-well migration as read-out, because the migration ability of MDA-MB-231 cells also depends on the presence of TNF and other cytokines in the surrounding environment^29^. DHTS treated medium strongly inhibited MDA-MB-231 migration more effectively than HuR depletion, whereas the DHTS treatment in siHuR cells produced an almost chemotactic inactive medium (Fig. 7E, left panel). Once again, HuR over-expression completely rescued DHTS efficacy and the corresponding medium was equally effective as the control medium. Notably, HuR over-expression *per se* did not produce an increased chemotactic medium (Fig. 7E, right panel). Taken all together, these data show that HuR completely rescues at least three phenotypic effects of DHTS, such as direct viability and proliferation inhibition on cancer cells and autocrine/paracrine inhibition of cancer cell migration. This suggests that HuR is a pivotal intracellular target of DHTS and that multi-target effects of DHTS, and eventually of tanshinones, can be explained by the inhibition of RNA-binding activity of HuR.

## Discussion

In this study we show that DHTS is a potent inhibitor of the HuR:RNA interaction, active in the low nanomolar range, mainly by limiting the association rate of HuR with RNA. This inhibition is functionally recapitulated in our cellular models, in which the known, DHTS induced down-regulation of TNF expression can be largely ascribed to the loss of function of HuR, that no longer stabilizes TNF mRNA neither mediates its polysomal loading. This molecular mechanism of action has a therapeutic relevance, as shown by the inhibitory effect on viability, proliferation and chemotaxis of breast cancer cell lines and by the decreased TNF production in macrophages cells. Remarkably, the molecular and phenotypic effects induced by short-term and low doses of DHTS in breast cancer cell lines are rescued by the over-expression of HuR, confirming the cellular interaction between these two molecules. HuR over-expression in cancer tissues and the mechanistic role in mediating the inflammatory process has suggested that its inhibition could be beneficial in these pathologies^22^,^30^,^31^. In addition, HuR has been proposed as a drug target in cardiovascular diseases, nephropathy and diabetic retinopathy^32^,^33^,^34^. Several *in vitro* studies^14^,^23^,^35^ introduced some naturally occurring small molecule as HuR inhibitors, however the correlation among their post-transcriptional mechanism, biological effect and specificity, and therapeutic usefulness remains elusive. The most noteworthy example is the naphthofuranone MS-444, that was identified by a screening campaign on ≈50,000 natural product extracts using confocal fluctuation spectroscopic assays^14^. MS-444 has been shown to inhibit the HuR:RNA interaction by blocking the dimerization of HuR upon binding to the target RNA with a K_d_ around 40 nM. We did not investigate if DHTS acts via a similar inhibition of HuR dimerization. MS-444 has been recently used as a mechanistic tool to prevent HuR binding to miR-16^36^ and to TDP-43 and FUS mRNA^37^, although at higher concentrations than DHTS. In a recent effort compounds with a coumarin-derived core, which interfere with the function of HuR in the nanomolar range, have been identified using fluorescence polarization. This class of compounds shows anticancer properties in cell lines by inhibition of the expression of anti-apoptotic HuR targets, such as Bcl-2, Msi1 and XIAP^38^. In our case, having RNA-binding activity as functional read-out, we measured a strong, nanomolar inhibition of the association rate constant between HuR and RNA *in vitro* that was also specific to HuR and not to other RBPs as Lin28b, TDP-43 and TTP. In particular, we observed a ≈60% reduction in the number of TNF mRNA copies, as well as, with different extent, for ERBB2, VEGF, and CCND1 transcripts, using 1 μM of DHTS by RIP experiments. Consistently, pull down experiments confirmed this effect although the magnitude of the interference was smaller (~40%), being limited by the use of a competitive exogenous RNA probe. The inhibitory effect of DHTS on AUF1 protein upon stimulation with LPS (Fig. 4B) could be ascribed to different mechanisms such as a diminished affinity for RNA or to an increased affinity for DHTS due to post-translational modifications, therefore we can not exclude a multi-targeting effect of DHTS in this condition. We did not observe a DHTS-induced activation of the p38 MAPK pathway nor HuR localization to the cytoplasm, further supporting the DHTS-induced HuR inhibition within cells and the utilization of DHTS in those cancer where HuR cytoplasmic localization plays a relevant role^31^. DHTS belongs to a family of natural diterpenes called tanshinones. Their anti-inflammatory, anti-atherosclerosis, cardioprotective and anti-cancer properties have been exploited in traditional Chinese medicine and are now under clinical investigation, but their exact mechanism of action is still unclear^17^,^39^. In this context we disclose a previously unrecognized molecular mechanism of action, involving post-transcriptional regulation, that might contribute to explain the wide spectrum of activities of DHTS correlated with their well-known inhibition of TNF. In particular, tanshinone I inhibits growth, invasion and angiogenesis on human breast cancer cells MDA-MB-231, both *in vitro* and *in vivo*, by decreasing the TNF-induced VEGF production. Moreover it reduces the MDA-MB-231 adhesion properties by decreasing the TNF dependent pivotal intercellular adhesion molecule-1 (ICAM-1) and vascular adhesion molecule-1 (V-CAM) of endothelial cells^29^. The efficacy of tanshinones has also been related with reduced expression of interleukins such as *IL-6*^18^, *MMPs*^40^, *VEGF*^41^ and *COX-2*^42^. There are also indications that the modulatory properties on an inflammatory state upon administration of tanshinones occurs via post-transcriptional repression of specific miRNAs, as in the case of miRNA-155 in colon cancer cells^43^. Interestingly, many of these key mRNAs and miRNAs are post-transcriptionally regulated by HuR^8^,^20^,^44^,^45^,^46^,^47^. Tanshinones have been shown to target or modulate several transcription factors, ion channels or hormone receptors within the cell^39^. We show that an intriguing explanation to the multi-target spectrum of tanshinones, could rely on the inhibition of the post-transcriptional function of HuR. Importantly resveratrol that has been shown to post-transcriptionally modulate TNF through KSRP regulation, providing a valuable example of the importance of post-transcriptional modulation of mRNA processing in the control of inflammation^16^. We provide mechanistic data indicating that HuR-mediated post-transcriptional inhibition is a major component of the cellular response to DHTS and that its relevance is shown by the HuR dosage modulation of cytotoxicity and migratory potential in breast cancer cells in response to DHTS. These findings suggest several important avenues for further research: (i) *HuR-dependent regulation of TNF expression levels*.Modulation of cytokines is a validated therapeutic strategy for the treatment of inflammatory disorders, and due to its predicted post-transcriptional regulation, it might be expected that administration of DHTS could attenuate the TNF protein levels rather than deplete TNF at systemic levels, such as a consequence of antibody-based therapeutic strategies^48^; (ii) *DHTS as a tool to investigate the connections between cancer and inflammation based on modulation of post-transcriptional events*.Once validated *in vivo*, the use of DHTS (or of some of its analogues with a better drug-like profile) for therapeutic purposes could be conceived. A functional screening of anti-inflammatory agents allowed the identification of 15,16-dihydrotanshinone-I (DHTS) for its ability to inhibit a specific protein-RNA interaction. First, we have characterized the biochemical parameters of DHTS in virtue of its interference on the dynamic of HuR-RNA binding and anticipated a new molecular scaffold exerting a previous unrecognized bioactivity. Second, we have reported mechanistic evidences, in human tumor and mouse macrophages cell lines, suggesting HuR among the early intracellular targets of DHTS. The loss of function of HuR in response to this agent explains the modulation of the stability and translational efficiency of target mRNAs. Third, we have shown that phenotypic response, in terms of migration and sensitivity, of breast cancer cells to DHTS is remarkably influenced by HuR expression. Overall, these findings advance the understanding of contribution of post-transcriptional control in mediating anti-inflammatory and anti-cancer effects of a class of natural compounds and expand the concept of “genome druggability” by adding the post-transcriptional activity of the RNA binding protein HuR as feasible event that can occur during a pharmacological treatment. Finally, we suggest a novel rationale for the use of tanshinones in human diseases where HuR is deregulated or has prognostic significance, as breast, colon or ovarian cancers.

## Methods

### Cell culture

Human breast adenocarcinoma MCF-7 (ICLC; HTL95021), SKBR3 (ICLC; HTL03005), and MDA-MB-231 (ICLC; HTL99004) cell lines were cultured in standard DMEM medium and growth conditions. Stable HuR-silenced (siHuR) and scramble (SCR) MCF-7 cells were obtained by infection with HuR shRNA- (ELAVL1 MISSION, Sigma, TRCN0000285492) and control shRNA (plasmid 1864, Addgene)-containing lentiviral particles, respectively; clones were selected with 5 μg/ml puromycin. MCF-7 over-expressing (HuROE) cells were obtained by transient transfection with Lipofectamine 2000 (Life Technologies; 11668-019) of pCMV6-HuR vector^23^. For endogenous TNF, MCF-7 cells (5*10^4/well) were seeded in 6-well plates and grown under standard conditions for 24 h. Approximately 10^3 DH5α *E. coli* cells were then inoculated for overnight co-culture in DMEM without antibiotics. After PBS washing steps, MCF-7 cells were treated for 3 h in complete medium with DHTS or DMSO. RAW264.7 monocytes were grown as MCF-7 but in DMEM plus 0.1 mM non-essential amino acids mixture (Life Technologies, 11140).

### Compounds and primary antibodies

Anti-inflammatory drugs used in the screening were cherry picked from the Spectrum Collection (MicroSource Discovery). Dihydrotanshinone I (D0947), Tanshinone I (T5330), Tanshinone IIA (T4952), Cryptotanshinone (C5624), and actinomycin D (A9400) were purchased from Sigma and dissolved in ultrapure dimethylsulfoxide (DMSO, Amresco, N182) to 10 mM final concentration. List of antibodies: anti-HuR (sc-71290), anti-TNF (sc-1351), anti-Actin (sc-1616) from Santa Cruz Biotechnology; mouse anti-GAPDH (MAB374) from EMD/Millipore; anti-RPL26 (ab59567) and anti-6x His (ab1187) from Abcam; TTP antiserum (SAK21B) was a kind gift from Dr. A.R. Clark^49^ (Centre for Translational Inflammation Research, University of Birmingham, Birmingham, UK).

### Expression and purification of HuR isoforms

The full-lenght sequence of HuR (NP_001410.20) in pCMV-HuR recombinant vector^50^ was used as template for PCR using M1_M2 (5′-CCCGCATATGATGTCTAATGGTTATG and 5′-TATACTCGAGGCGAGAGGAGTGCC) and M2_M3 (5′- CCGCATATGATGACCCAGAAGGACGTA and 5′- GGCCTCGAGTTTGTGGGACTTGT) primers. Inserts were sub-cloned in NdeI/XhoI-digested pET-42a vector (kindly provided by dr. Filipowicz’s lab, FMI, Basel, Switzerland) to produce M1_M2 (aa 1 to 197; predicted molecular weight: 22 kDa) and M2_M3 (aa 117 to 326; predicted molecular weight: 24 kDa) His-tagged proteins, respectively. Sub-cloning of single domains was obtained with the following primers: M1 (5′-CCCGCATATGATGTCTAATGGTTATG and 5′-CCGCTCGAGTACGTCCTTCTGGG); M2 (5′-CCGCATATGATGACCCAGAAGGACGTA and 5′-GGCTCGAGTCGCGCTGGCGAGT); M3 (5′-GGCATATGTCCTCCGGCTGGTGCAT and 5′-GGCCTCGAGTTTGTGGGACTTGT). Protein expression and purifications were performed as already described^51^. Purity of eluates was evaluated by 15% SDS-PAGE and Coomassie staining; single bands were quantified using ImageJ 1.4 software (NIH) with respect to known amount of loaded BSA.

### AlphaScreen and electrophoresis mobility shift (REMSA) assays

Recombinant HuR-cMyc-His protein preparation, REMSAs with a fluorescent RNA probe (5′-Cy3-AUUAUUUAUUAUUUAUUUAUUAUUUA), AlphaScreen with a 5′-biotinylated RNA probe (BiTNF, 5′-AUUAUUUAUUAUUUAUUUAUUAUUUA), and screening assays were carried out as already described^23^. Equilibrium dissociation constants (Ki) of DHTS were fitted according to 1-site competition model in GraphPad Prism®, version 5.0 (GraphPad Software, Inc., San Diego, CA), keeping constant the RNA concentration (50 nM) and the K_d_ of the reaction at equilibrium (2.5 nM). IC_50_ values were obtained by nonlinear regression of log(dose)-response fit using the same software. Time course experiments were performed by reacting ligands and DHTS simultaneously, or by pre-incubating DHTS with rHuR or RNA. Dissociation experiments were performed upon 30 min pre-incubation of 1 nM of rHuR and 50 nM of RNA plus beads (“Ligands+beads” in Fig. 1G), before addition of DHTS. To exclude possible interference of beads on dissociation kinetics protein and RNA (“Ligands” in Fig. 1G) were pre-incubated for 30 min, then DHTS, at the indicated concentrations, and, finally, beads were added. Curves were fitted according to the kinetics of competitive binding model in GraphPad software, keeping constant the k_on_ (2.76 ± 0.56*10^6 M^–1^ min^–1^) and k_off_ (0.007 ± 0.005 min^–1^) of the reaction. DHTS analogs were tested by REMSA at the indicated concentrations and at equilibrium^52^. Plasmids encoding recombinant Lin28b-cMyc-His, TDP-43-His, and HuD-His were kindly provided by Prof. Quattrone’s lab (CIBIO, University of Trento, Italy). TTP, Lin28b, and HuD were expressed in HEK293T cells and purified following the protocols reported in^23^, with exception of buffers for Lin28b purification that were supplemented with 15 μM ZnCl_2_. REMSAs were performed using the fluorescent AU-rich RNA probe to test TTP and HuD protein activities, whereas Lin28b binding was tested against pre-let7g (5′-Cy3-GUCUAUGAUACCACCCGGUACAGGAGAU)^53^ and TDP-43 against the 5′-Cy3-CCGGGGCCGGGGCCGGGGCCGGGG RNA probes. Further details about biochemical experiments in the supplemental information.

### Nucleo-cytoplasmic cell fractionation and qPCR

For nucleo-cytoplasmic fractionation, cells (4–6*10^6/sample) were re-suspended in buffer C (20 mMTris-HCl pH 7.5, 75 mM NaCl, 5 mM MgCl_2_, 0.5% p/w sodium deoxycholate, 0.2% Triton, 1 mM DTT, 0.5% glycerol) plus protease inhibitor cocktail (Sigma, P8340) and 1 U/μl RNAse inhibitor (Thermo Scientific, EO0381). After centrifugation supernatants were collected (cytoplasmic lysates), whereas pelleted nuclei were re-suspended in buffer N (10 mM Tris-HCl pH 8, 25 mM NaCl, 5 mM MgCl_2_, 1% p/w sodium deoxycholate, 1% Triton, 0.2% SDS, 1 mM DTT) plus protease and RNAse inhibitors and sonicated as above. TRIzol reagent (Life Technologies, 12183-555) was used for RNA isolation. Quantitative PCRs, after cDNA Synthesis (Thermo Scientific, K1612) with equimolar mix of random and oligo-dT primers and two micrograms of template RNA, were performed using Universal SYBR Master Mix (KAPA Biosystems, KR0389) on CFX-96/384 thermal cyclers (BIO-RAD). 2^−ΔΔCt^ method was used for quantification of mRNAs. Forward and reverse primers used: RNA18S5 (GCAGCTAGGAATAATGGAATAG and TGGCAAATGCTTTCGCTCTG), TNF (5′-GGGACCTCTCTCTAATCAGC and 5′-TCAGCTTGAGGGTTTGCTAC), GAPDH (5′-CAAGGTCATCCATGACAACTT and 5′-GTCCACCACCCTGTTGCTGTA), CD14 (5′-GAAGCT AAAGCACTTCCAGAGC and 5′-TTCATCGTCCAGCTCACAAG), ERBB2 (GGTACTGAAAGCCTTAGGGAAGC and ACACCATTGCTGTTCCTTCCTC), VEGF (CCGCAGACGTGTAAATGTTCCT and CGGCTTGTCACATCTGCAAGTA), and CCND1 (CAGAACACGGCTCACGCTTAC and CTTGCCCCATCACGACAGAC). Primers for pre-TNF, pre-BRCA1, pre-CTCF, pre-MDM2, pre-MYBL2, and pre-NFATC3 are described elsewhere^28^.

### Polysomal profiling and protein/RNA isolation

For polysomal RNA profiling, cytoplasmic lysates of 2*10^7 MCF-7 cells/sample were subjected to 15–50% sucrose gradient ultracentrifugation and fractionation following reported protocols^6^,^54^,^55^. Aliquots of cytoplasmic lysates were considered for normalization. From each sub-polysomal or polysomal fraction, protein samples were obtained by precipitation with 10% final concentration of trichloroacetic acid (TCA), while RNAs were isolated by TRIzol reagent (1:5 v/v). Fractions 1 to 6 were pooled to represent sub-polysomal RNA samples, while pooling of fractions 7 to 12 represented polysomal RNA samples.

### RNA immunoprecipitation (RIP) and RNA pull-down assays

Five*10^6 cells/sample were used for each RIP experiment, performed as described in^56^ without cross-linking steps and using 0.8 μg/ml of anti-Hu antibody or of mouse IgG isotype (negative control). TRIzol reagent was added directly to the beads for HuR-bound RNA isolation. Fold enrichment was calculated as 2e-ΔCt, ΔCt = target mRNA IP HuR/(target mRNA IgG). For RNA pull-down assays, MCF-7 cells were lysed in buffer R (20 mM HEPES pH 7.5, 50 mM KCl, 0.5 μg BSA, 0.25% Glycerol, + protease and RNAse inhibitors) by sonication (80 amplitude with 6–7 cycles of 7” *on* and 45” *off*) at 4 °C. Clear lysates (0.2 μm-filtered) were incubated for 1 h at 4 °C with 0.5 μM of positive (BiTNF) or negative biotinylated (BiTNFneg, 5′-ACCACCCACCACCCACCCACCACCCA) RNA probes^23^. Solutions were incubated for further 2 h with 30 μl/samples of streptavidin magnetic beads (Life technologies, 11205D). Specific protein enrichments in beads-precipitated samples were analyzed by immunoblotting and densitometric analysis obtained using Image J 1.4 software (NIH).

### RNA stability assays

SCR, siHuR, vector, and HuROE MCF-7 cells were co-treated with Act-D (2 μM) and DMSO or DHTS for 3 h. Kinetics for mRNA stability evaluation has been carried out by extracting RNA in five time points (0, 30, 60, 120, 240 min) to be used for cDNA synthesis and quantitative PCR analyses. Residual levels of target mRNAs were normalized to those of RNA18S5, and data were plotted as function of time with respect 0 min condition.

### Cytotoxicity, Click-iT and RTCA assays

Sensitivity to DHTS was evaluated by CellTiter-Glo® (Promega, G7570), alamarBlue or MTT reagents following suggested protocols.

Ectopic expression of human TNF has been obtained with the pUNO1-hTNFA (Invivogen) plasmid. Inserts for recombinant pUNO-hTNFA/3′UTR and pUNO-hTNFA/3′UTRΔARE plasmids have been obtained by digestion of pBS vectors^57^,^58^, respectively, with XbaI and then blunt-end ligated in pUNO1-hTNFA vector digested with NheI restriction enzyme. Sequencing confirmed the results.

Apoptosis was evaluated by Caspase-Glo® 3/7 luminescent assay (Promega, G8090) upon normalization to the number of trypan blue negative cells. RNA transcription was assessed using Click-iT® RNA Alexa Fluor® 488 Imaging Kit (Life Technologies, C10330). EthynylUridine (EU) was added 30 min before fixation, permeabilization and Click-iT reaction. Fluorescence signals relative to nascent RNA and nuclei (Hoechst 33342) were detected with Operetta instrument (PerkinElmer)and analyzed with Harmony 3.5.2 software (PerkinElmer). Proliferation assays were carried out with the xCELLigence RTCA DP Instrument (Roche) by plating 5,000 cells/well at t_0_ in E-Plate-16 format. Parallel plates were used to check the magnitude of HuR silencing or over-expression. Migration assays were performed using the same instrument and settings for CIM-Plates-16 (Roche), using media (1% FBS) of MCF-7 cells in the lower chamber and MDA-MB-231 cells equally seeded (20,000/well/160 μl) in the upper chamber.

### Immunoblotting and Enzyme-linked immunosorbent (ELISA) assay

Total, nuclear, and cytoplasmic cell extracts were subjected to 15%-SDS-PAGE and resolved proteins were transferred to nitrocellulose membrane (Millipore, IPVH00010) as previously described^59^. ELISA assays were carried out using Human TNF kit (Thermo Scientific, EH3TNFA) and the suggested protocol using surnatants of 95%-confluent MCF-7 cells at time of treatment, seeded in 12-well plates.

### Statistical analysis

All data are expressed as means ± SD from three to four independent experiments and statistics was performed using one-way ANOVA with Bonferroni’s multiple comparison test and Alpha level of 0.05. Magnitude of significance was also evaluated by student *t-test* and probability (P) values <0.05, <0.01, and <0.001 were indicated with *, **, *** symbols, respectively.

## Additional Information

**How to cite this article**: D’Agostino, V. G. *et al*.Dihydrotanshinone-I interferes with the RNA-binding activity of HuR affecting its post-transcriptional function. *Sci. Rep*.**5**, 16478; doi: 10.1038/srep16478 (2015).

## Supplementary Material

Supplementary Information

Supplementary Table S1
