# Supplementary material for: Dihydrotanshinone-I interferes with the RNA-binding activity of HuR affecting its post-transcriptional function
Source: Sci Rep. 2015 Nov 10;5:16478. doi: 10.1038/srep16478 (PMC4639722; doi:10.1038/srep16478)
Supplement: Supplementary Information [file srep16478-s1.pdf]

Dihydrotanshinone-I interferes with the RNA-binding activity of HuR affecting its post-transcriptional function

Vito Giuseppe D'Agostino, Preet Lal, Barbara Mantelli, Christopher Tiedje, Chiara Zucal, Natthakan Thongon, Matthias Gaestel, Elisa Latorre, Luciana Marinelli, Pierfausto Seneci, Marialaura Amadio and Alessandro Provenzani

SUPPLEMENTAL FIGURES AND LEGENDS

Figure S1

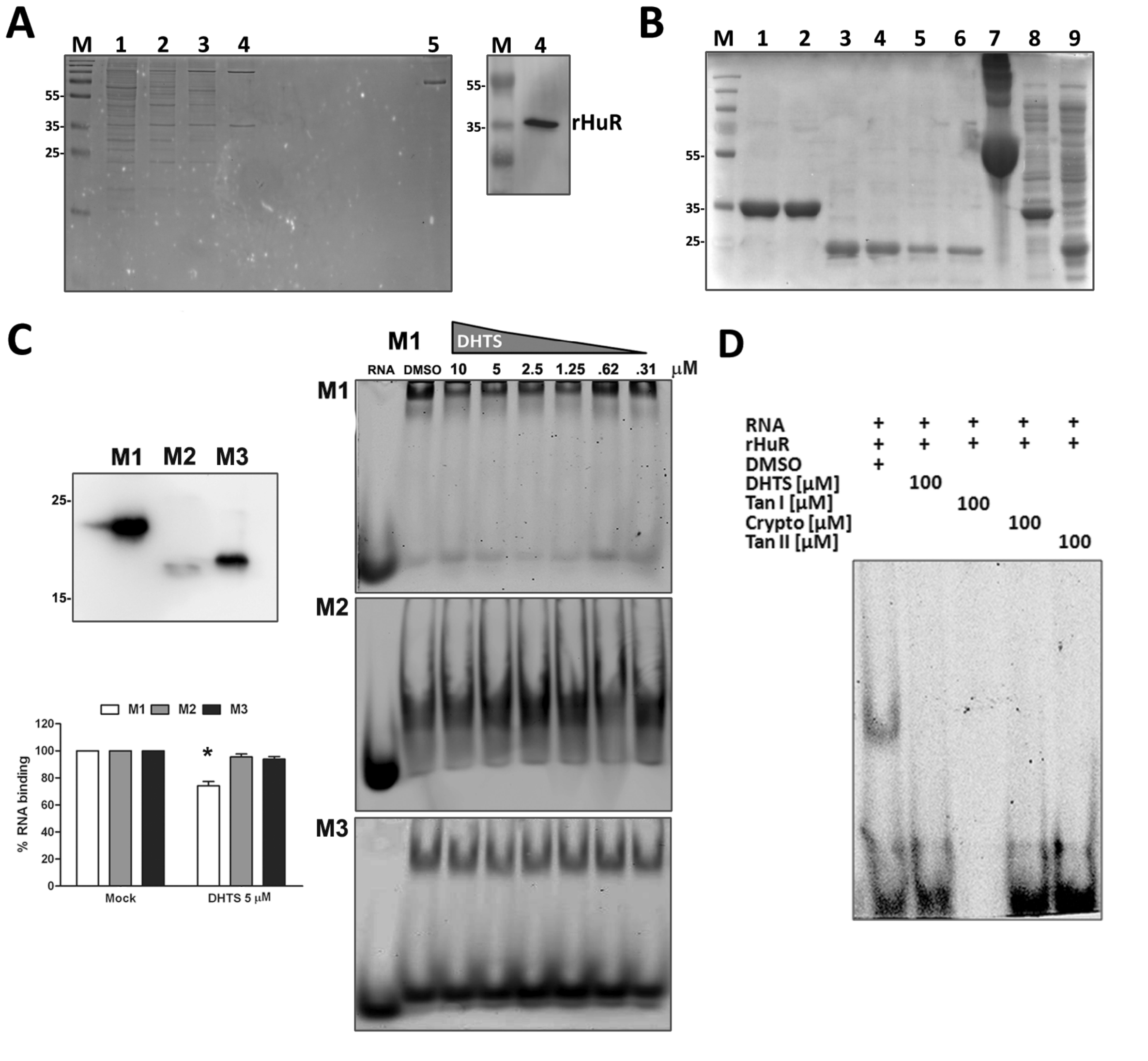

Figure S1. **Purification of HuR recombinant proteins and REMSA testing DHTS analogs.** (A) Coomassie staining (left side) or western blot (right side) of purified rHuR. M: marker, 1: crude

pre-cleared HEK293T protein extract, 2-4: elution of rHuR with 100-500 mM Imidazole gradient, 5: BSA. (B) Coomassie staining on 15%SDS PAGE. Purification of *E. coli* expressed HuR full-lenght (1-2), M1_M2 (3-4) and M2_M3 (5-6) isoforms; BSA (7), full-lenght (8) and M1_M2 (9) crude extracts. (C) Western blot using anti-His(6x) primary antibody showing size of purified HuR M1, M2, and M3 domains (top left). REMSAs indicate the activity of each single protein domain in presence or not of different doses of DHTS compound. Graph of densitometric and statistical analysis represents mean and SD on three independent experiments (*P value<0.05). (D) REMSA performed with 0.5 μM of rHuR and 0.5 μM of Cy-3 RNA probe at equilibrium showing the inhibitory activity of tanshinones against the HuR:RNA complex formation at 100 μM.

Figure S2

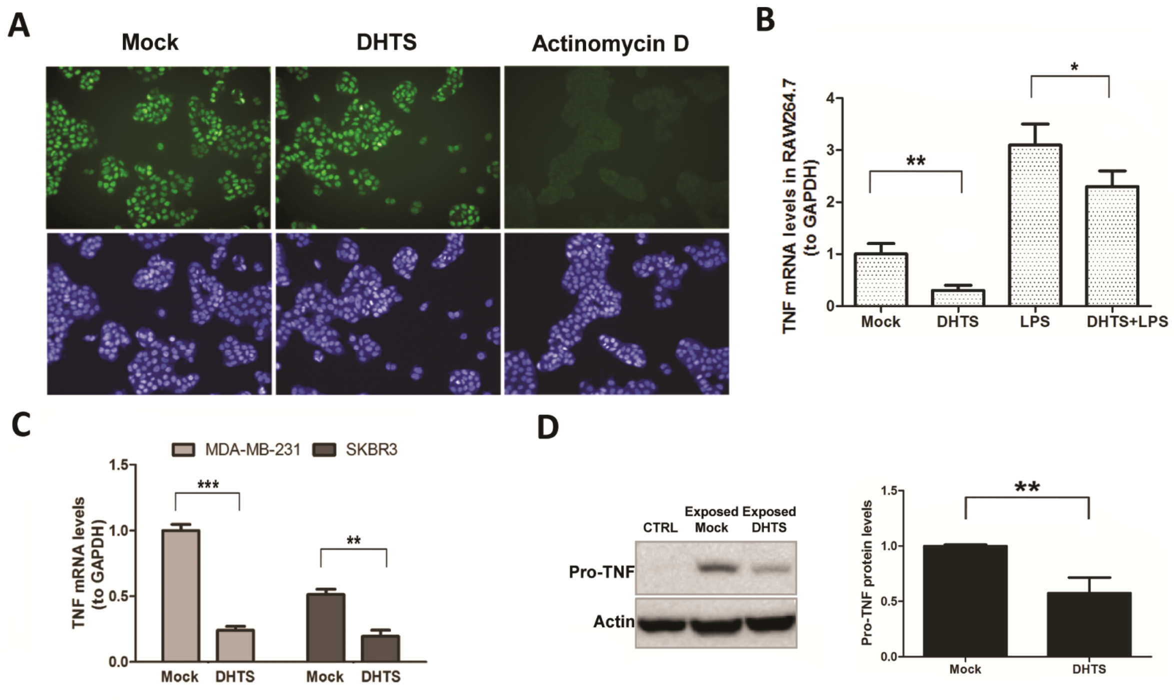

Figure S2. **Evaluation of global transcription in MCF-7 cells, of TNF mRNA expression in RAW264.7 and human breast cancer cells, and of pro-TNF levels in MCF-7 cells.** (A) Representative images of MCF-7 in Click-iT assays with incorporated EU (Alexa-488, green) and nuclei staining with Hoechst 3342 (blue). (B) Q-PCR on mouse TNF mRNA normalized with GAPDH mRNA on RAW264.7 cells treated with DMSO or 1 µM of DHTS for 3 h. (C) Q-PCR on TNF mRNA normalized with GAPDH mRNA levels on two different breast cancer cell lines treated with DMSO or 1 µM of DHTS for 3 h. (D) Endogenous human pro-TNF protein levels of *E. coli* cells un-exposed (CTRL) or overnight exposed MCF-7,treated with 1 µM of DHTS for 3 h or un-treated. Mean±SD refers to three independent experiments (n=3).

Figure S3

A

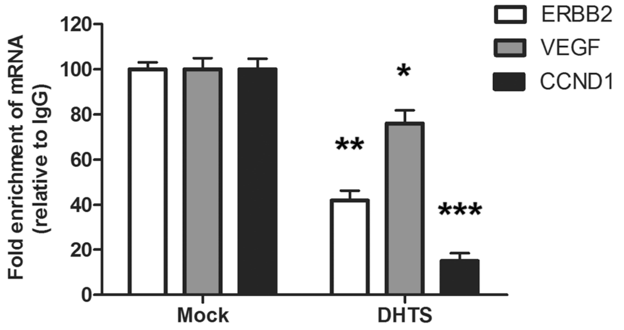

B

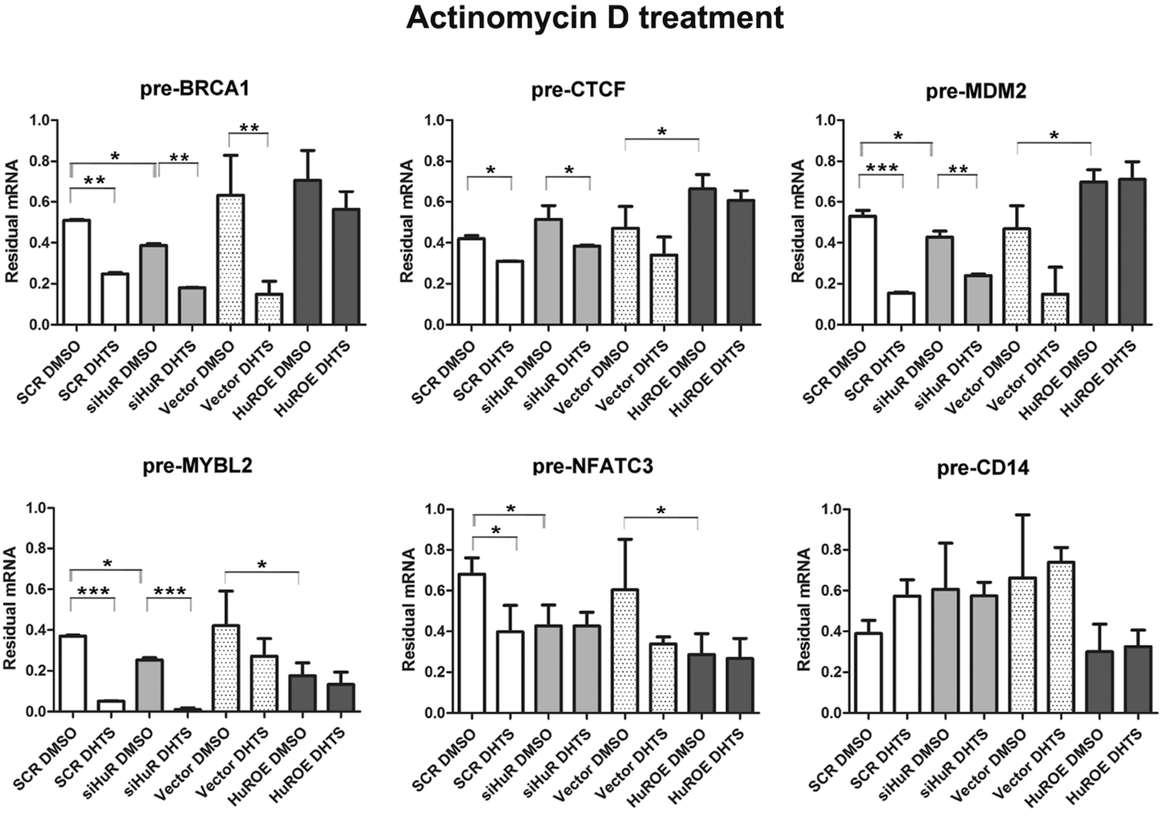

Figure S3. **DHTS inhibits intracellular association of HuR with other target mRNAs and influences stability of pre-mRNAs.** (A) RNA immunoprecipitation and Q-PCR analyses, by normalizing on IgG relative levels, of ERBB2, VEGF, and CCND1 mRNAs upon treating or not MCF-7 cells with 1 μM of DHTS for 3 h. (B) mRNA stability evaluation (as in Fig. 4) after 3 h actinomycin D treatment of scramble, vector, HuR silenced (siHuR) and HuR over-expressing (HuROE) MCF-7 cells. Residual mRNA, normalized to relative GAPDH mRNA levels, was

calculated with respect to t_0 after 60 min Mean±SD refers to three independent experiments (n=3).

Figure S4

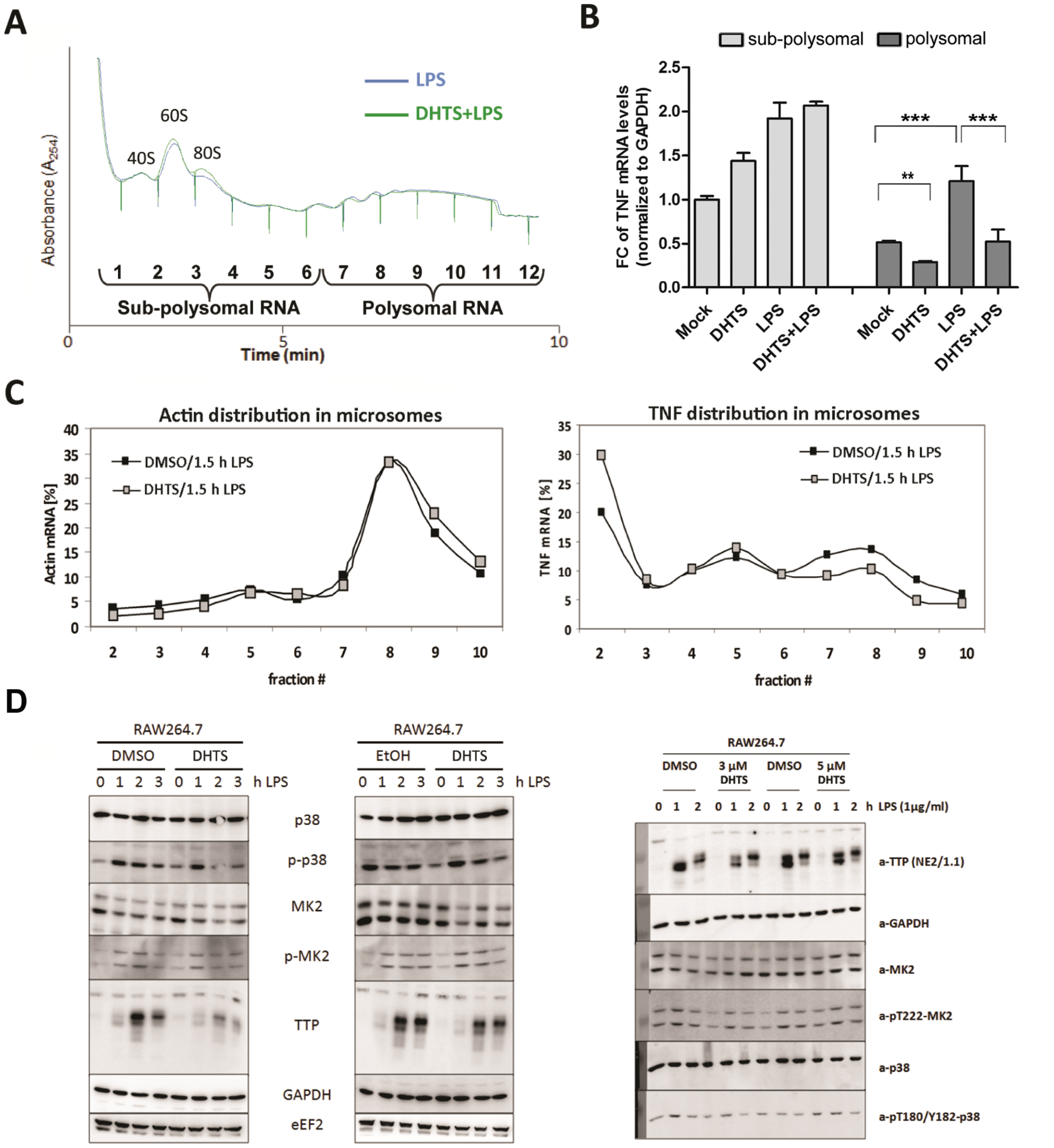

Figure S4. Polysomal profiles of MCF-7 and distribution of TNF mRNA in fractionated RNAs of RAW264.7 cells; effect of DHTS on p38 MAPK pathway. (A) Polysomal profiles of MCF-7 obtained as in Figure 5B. (B) Q-RT-PCR analysis of GAPDH and TNF mRNA levels in pooled sub-polysomal or polysomal RNA fractions. (C) Examples of polysomal distribution of TNF and Actin mRNAs of LPS-activated RAW264.7 cells treated or not with 0.5 µM of DHTS for 3 h. Distribution of TNF and as a control β-Actin mRNA in the fraction was analysed by Q-RT-

PCR as described previously [7]. DHTS led to a reduced association of TNF mRNA with polysomal fractions. (C). (D). Pre-incubation of RAW264.7 cells for 3 h with different amounts of DHTS dissolved in DMSO or absolute Ethanol and consequent LPS-activation for different times did not affect proper activation of the p38 MAPK pathway as indicated by the LPS-induced phosphorylation of p38 at T180/Y182 and its downstream-activated kinase MK2 at T222. In addition, expression of pro-inflammatory TTP was strongly induced and not altered, too.

SUPPLEMENTAL TABLE

See excel file: Table S1

Table S1. List of 107 anti-inflammatory compounds screened for in vitro inhibition of the rHuR:RNA interaction, ordered according to Z-score. The original position of each compound in the plate is reported in column A.
